# 1,8-Dihydr­oxy-2,4,5,7-tetra­nitro-9,10-anthraquinone

**DOI:** 10.1107/S1600536810014431

**Published:** 2010-04-24

**Authors:** Mahsa Armaghan, Mostafa M. Amini, Seik Weng Ng

**Affiliations:** a Department of Chemistry, General Campus, Shahid Beheshti University, Tehran 1983963113, Iran; b Department of Chemistry, University of Malaya, 50603 Kuala Lumpur, Malaysia

## Abstract

The ring system in the title compound, C_14_H_4_N_4_O_12_, is essentially planar (r.m.s. deviation of the carbon atoms = 0.085 Å); the two hydr­oxy groups form intra­molecular hydrogen bonds to the same carbonyl O atom. The nitro groups are twisted with respect to the mean plane of the ring system by 74.3 (1) (1-nitro), 42.3 (3) (3-nitro), 45.7 (3) (6-nitro) and 66.9 (1)° (8-nitro).

## Related literature

For the synthesis of the title compound, see: Teich *et al.* (2004 ▶). For related structures, see: Armaghan *et al.* (2010 ▶); Brown & Colclough (1983 ▶), Yatsenko *et al.* (1996 ▶).
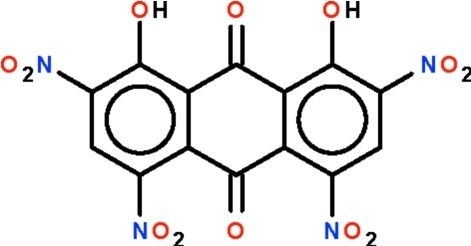


## Experimental

### 

#### Crystal data

C_14_H_4_N_4_O_12_
*M* *_r_* = 420.21
Monoclinic, 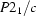

*a* = 17.726 (2) Å
*b* = 9.007 (1) Å
*c* = 9.731 (1) Å
β = 102.643 (2)°
*V* = 1515.9 (3) Å^3^
*Z* = 4
Mo *K*α radiation
μ = 0.17 mm^−1^
*T* = 223 K
0.35 × 0.25 × 0.10 mm

#### Data collection

Bruker SMART APEX diffractometer
11323 measured reflections
2672 independent reflections
2034 reflections with *I* > 2σ(*I*)
*R*_int_ = 0.045

#### Refinement

*R*[*F* ^2^ > 2σ(*F* ^2^)] = 0.063
*wR*(*F* ^2^) = 0.215
*S* = 1.10
2672 reflections
279 parameters
2 restraints
H atoms treated by a mixture of independent and constrained refinement
Δρ_max_ = 0.35 e Å^−3^
Δρ_min_ = −0.36 e Å^−3^

### 

Data collection: *APEX2* (Bruker, 2009 ▶); cell refinement: *SAINT* (Bruker, 2009 ▶); data reduction: *SAINT*; program(s) used to solve structure: *SHELXS97* (Sheldrick, 2008 ▶); program(s) used to refine structure: *SHELXL97* (Sheldrick, 2008 ▶); molecular graphics: *X-SEED* (Barbour, 2001 ▶); software used to prepare material for publication: *publCIF* (Westrip, 2010 ▶).

## Supplementary Material

Crystal structure: contains datablocks global, I. DOI: 10.1107/S1600536810014431/bt5249sup1.cif

Structure factors: contains datablocks I. DOI: 10.1107/S1600536810014431/bt5249Isup2.hkl

Additional supplementary materials:  crystallographic information; 3D view; checkCIF report

## Figures and Tables

**Table 1:** Hydrogen-bond geometry (Å, °)

*D*—H⋯*A*	*D*—H	H⋯*A*	*D*⋯*A*	*D*—H⋯*A*
O1—H1⋯O2	0.84 (3)	1.84 (3)	2.579 (3)	146 (5)
O3—H3⋯O2	0.84 (4)	1.82 (3)	2.576 (3)	148 (5)

**Fig. 1.**
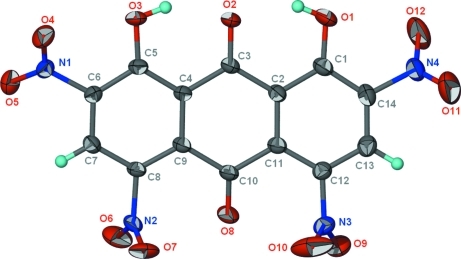
Anisotropic displacement ellipsoid plot (Barbour, 2001) of C14H4N4O12; ellipsoids are drawn at the 50% probability level and H atoms are of arbitrary radius.

## supplementary crystallographic information

### Comment

In continuation to our previous synthesis of anthraquinone derivatives for the absorption of aromatic sulfur compounds from oil when immobilized on silica surface (MCM-41) (Armaghan *et al.*, 2010), we have synthesized the title compound. The compound was reported in a previous report (Teich *et al.*, 2004). In the present study, the synthesis involves functionalization of 1,8-dihydroxy-anthraquinone with the fuming nitric acid. The compound (Scheme I, Fig. 1) is soluble in methanol.

### Experimental

Fuming nitric acid (4 ml) was added to a solution of 1,8-dihydroxy-anthraquinone (240 mg, 1.0 mmol) dissolved in concentrated sulfuric acid (5 ml). The mixture was stirred for 2 hours. It was then poured into ice (100 g). The yellow precipitate was washed with water. Crystals were obtained by   slow diffusion of *n*-hexane into a methanol solution of the title compound; m.p.> 473 K.

### Refinement

Carbon-bound H-atoms were placed in calculated positions (C—H 0.94 Å) and were included in the refinement in the riding model approximation, with *U*(H) set to 1.2*U*(C). The oxygen-bound H-atoms were located in a difference Fourier map. They were refined isotropically with a distance restraint of O–H 0.84±0.01 Å.

The parameters in the weighting scheme are somewhat large; these could not be reduced without affecting the goodness of fit.

### Crystal data

**Table:**

C_14_H_4_N_4_O_12_	*F*(000) = 848
*M**_r_* = 420.21	*D*_x_ = 1.841 Mg m^−^^3^
Monoclinic, *P*2_1_/*c*	Mo *K*α radiation, λ = 0.71073 Å
Hall symbol: -P 2ybc	Cell parameters from 2323 reflections
*a* = 17.726 (2) Å	θ = 2.3–27.5°
*b* = 9.007 (1) Å	µ = 0.17 mm^−^^1^
*c* = 9.731 (1) Å	*T* = 223 K
β = 102.643 (2)°	Plate, brown
*V* = 1515.9 (3) Å^3^	0.35 × 0.25 × 0.10 mm
*Z* = 4	

### Data collection

**Table:**

Bruker SMART APEX diffractometer	*R*_int_ = 0.045
graphite	θ_max_ = 25.0°, θ_min_ = 1.2°
ω scans	*h* = −19→21
11323 measured reflections	*k* = −10→10
2672 independent reflections	*l* = −11→11
2034 reflections with *I* > 2σ(*I*)	

### Refinement

**Table:**

Refinement on *F*^2^	Primary atom site location: structure-invariant direct methods
Least-squares matrix: full	Secondary atom site location: difference Fourier map
*R*[*F*^2^ > 2σ(*F*^2^)] = 0.063	Hydrogen site location: inferred from neighbouring sites
*wR*(*F*^2^) = 0.215	H atoms treated by a mixture of independent and constrained refinement
*S* = 1.10	*w* = 1/[σ^2^(*F*_o_^2^) + (0.137*P*)^2^ + 0.3859*P*] where *P* = (*F*_o_^2^ + 2*F*_c_^2^)/3
2672 reflections	(Δ/σ)_max_ = 0.001
279 parameters	Δρ_max_ = 0.35 e Å^−^^3^
2 restraints	Δρ_min_ = −0.36 e Å^−^^3^

### Fractional atomic coordinates and isotropic or equivalent isotropic displacement parameters (Å^2^)

**Table:**

	*x*	*y*	*z*	*U*_iso_*/*U*_eq_	
O1	0.34937 (16)	0.2030 (3)	0.4649 (3)	0.0528 (7)	
H1	0.323 (2)	0.172 (5)	0.388 (3)	0.082 (16)*	
O2	0.24924 (13)	0.2174 (2)	0.2291 (2)	0.0417 (6)	
O3	0.15091 (14)	0.2212 (2)	−0.0089 (3)	0.0410 (6)	
H3	0.183 (2)	0.186 (6)	0.060 (4)	0.099 (18)*	
O4	0.07048 (14)	0.2831 (3)	−0.2661 (3)	0.0503 (7)	
O5	−0.02307 (13)	0.4233 (3)	−0.2381 (3)	0.0465 (7)	
O6	0.06528 (16)	0.8754 (3)	0.0919 (3)	0.0606 (8)	
O7	0.17014 (16)	0.9106 (3)	0.0217 (3)	0.0565 (8)	
O8	0.22018 (17)	0.7979 (3)	0.2950 (3)	0.0614 (9)	
O9	0.33793 (14)	0.8827 (3)	0.5708 (3)	0.0507 (7)	
O10	0.3791 (2)	0.8835 (3)	0.3801 (4)	0.0895 (12)	
O11	0.51679 (18)	0.4003 (4)	0.7123 (4)	0.0879 (12)	
O12	0.43047 (19)	0.2420 (4)	0.7288 (3)	0.0806 (11)	
N1	0.04326 (15)	0.3791 (3)	−0.2055 (3)	0.0333 (6)	
N2	0.12252 (16)	0.8324 (3)	0.0558 (3)	0.0348 (6)	
N3	0.35601 (16)	0.8218 (3)	0.4727 (3)	0.0413 (7)	
N4	0.45331 (17)	0.3494 (3)	0.6774 (3)	0.0438 (7)	
C1	0.34783 (18)	0.3492 (4)	0.4612 (3)	0.0348 (7)	
C2	0.29723 (16)	0.4320 (3)	0.3556 (3)	0.0293 (7)	
C3	0.24621 (16)	0.3536 (3)	0.2380 (3)	0.0305 (7)	
C4	0.19253 (16)	0.4400 (3)	0.1305 (3)	0.0279 (7)	
C5	0.14731 (17)	0.3649 (3)	0.0136 (3)	0.0292 (7)	
C6	0.09371 (17)	0.4510 (3)	−0.0830 (3)	0.0295 (7)	
C7	0.08449 (17)	0.5998 (3)	−0.0660 (3)	0.0303 (7)	
H7	0.0465	0.6532	−0.1295	0.036*	
C8	0.13215 (16)	0.6704 (3)	0.0466 (3)	0.0284 (7)	
C9	0.18590 (16)	0.5934 (3)	0.1447 (3)	0.0270 (7)	
C10	0.23464 (18)	0.6727 (3)	0.2673 (3)	0.0326 (7)	
C11	0.29712 (17)	0.5868 (3)	0.3616 (3)	0.0296 (7)	
C12	0.35047 (17)	0.6585 (4)	0.4664 (3)	0.0324 (7)	
C13	0.40121 (18)	0.5807 (4)	0.5685 (3)	0.0374 (8)	
H13	0.4371	0.6309	0.6386	0.045*	
C14	0.39833 (18)	0.4294 (4)	0.5659 (3)	0.0374 (8)	

### Atomic displacement parameters (Å^2^)

**Table:**

	*U*^11^	*U*^22^	*U*^33^	*U*^12^	*U*^13^	*U*^23^
O1	0.0710 (18)	0.0263 (13)	0.0517 (16)	0.0112 (11)	−0.0070 (13)	0.0058 (11)
O2	0.0511 (14)	0.0192 (12)	0.0471 (14)	0.0043 (9)	−0.0062 (11)	0.0007 (9)
O3	0.0589 (16)	0.0178 (11)	0.0410 (13)	−0.0005 (10)	−0.0008 (11)	−0.0021 (9)
O4	0.0553 (16)	0.0469 (15)	0.0447 (14)	0.0009 (12)	0.0024 (11)	−0.0179 (12)
O5	0.0356 (13)	0.0538 (15)	0.0459 (14)	−0.0033 (11)	−0.0002 (10)	−0.0021 (11)
O6	0.0752 (19)	0.0413 (15)	0.0698 (19)	0.0242 (13)	0.0254 (15)	0.0017 (12)
O7	0.0714 (18)	0.0215 (12)	0.080 (2)	−0.0048 (12)	0.0241 (15)	0.0037 (12)
O8	0.094 (2)	0.0270 (14)	0.0471 (15)	0.0208 (13)	−0.0187 (14)	−0.0113 (11)
O9	0.0628 (16)	0.0403 (14)	0.0484 (15)	0.0035 (11)	0.0108 (12)	−0.0159 (11)
O10	0.174 (4)	0.0379 (16)	0.074 (2)	−0.0259 (18)	0.066 (2)	−0.0057 (14)
O11	0.057 (2)	0.092 (3)	0.097 (3)	0.0004 (17)	−0.0214 (18)	0.020 (2)
O12	0.075 (2)	0.089 (2)	0.067 (2)	0.0012 (17)	−0.0084 (16)	0.0411 (18)
N1	0.0357 (15)	0.0309 (14)	0.0308 (13)	−0.0076 (11)	0.0020 (11)	0.0005 (11)
N2	0.0458 (16)	0.0257 (14)	0.0300 (13)	0.0127 (12)	0.0018 (11)	0.0004 (11)
N3	0.0558 (17)	0.0317 (15)	0.0339 (15)	−0.0049 (13)	0.0047 (13)	−0.0061 (12)
N4	0.0417 (17)	0.0464 (18)	0.0391 (16)	0.0086 (13)	−0.0002 (13)	0.0057 (13)
C1	0.0400 (17)	0.0286 (17)	0.0348 (16)	0.0070 (13)	0.0064 (13)	0.0033 (12)
C2	0.0344 (16)	0.0266 (16)	0.0262 (15)	0.0051 (12)	0.0052 (12)	0.0020 (11)
C3	0.0357 (17)	0.0223 (17)	0.0321 (16)	0.0040 (11)	0.0045 (13)	0.0021 (12)
C4	0.0301 (15)	0.0205 (15)	0.0324 (15)	0.0006 (11)	0.0052 (12)	0.0015 (11)
C5	0.0361 (16)	0.0214 (15)	0.0307 (15)	−0.0014 (12)	0.0085 (12)	0.0007 (11)
C6	0.0315 (15)	0.0259 (16)	0.0297 (15)	−0.0030 (12)	0.0041 (12)	−0.0004 (12)
C7	0.0321 (16)	0.0291 (16)	0.0285 (15)	0.0032 (12)	0.0041 (12)	0.0043 (12)
C8	0.0348 (16)	0.0196 (15)	0.0310 (15)	0.0039 (12)	0.0078 (12)	0.0003 (12)
C9	0.0321 (15)	0.0218 (15)	0.0273 (15)	0.0037 (12)	0.0071 (12)	0.0019 (11)
C10	0.0457 (18)	0.0206 (16)	0.0294 (15)	0.0046 (13)	0.0034 (13)	0.0000 (12)
C11	0.0377 (17)	0.0241 (16)	0.0272 (15)	0.0027 (12)	0.0073 (13)	0.0002 (11)
C12	0.0380 (16)	0.0294 (17)	0.0295 (15)	−0.0012 (13)	0.0064 (12)	−0.0019 (12)
C13	0.0398 (18)	0.0413 (19)	0.0297 (16)	−0.0005 (14)	0.0047 (13)	−0.0039 (13)
C14	0.0355 (17)	0.042 (2)	0.0319 (17)	0.0073 (14)	0.0019 (13)	0.0037 (14)

### Geometric parameters (Å, °)

**Table:**

O1—C1	1.317 (4)	C1—C14	1.401 (4)
O1—H1	0.84 (3)	C1—C2	1.419 (4)
O2—C3	1.232 (4)	C2—C11	1.395 (4)
O3—C5	1.316 (4)	C2—C3	1.474 (4)
O3—H3	0.84 (4)	C3—C4	1.473 (4)
O4—N1	1.206 (3)	C4—C9	1.396 (4)
O5—N1	1.216 (3)	C4—C5	1.412 (4)
O6—N2	1.208 (4)	C5—C6	1.413 (4)
O7—N2	1.201 (4)	C6—C7	1.364 (4)
O8—C10	1.199 (4)	C7—C8	1.383 (4)
O9—N3	1.203 (4)	C7—H7	0.9400
O10—N3	1.205 (4)	C8—C9	1.379 (4)
O11—N4	1.194 (4)	C9—C10	1.493 (4)
O12—N4	1.199 (4)	C10—C11	1.491 (4)
N1—C6	1.475 (4)	C11—C12	1.389 (4)
N2—C8	1.474 (4)	C12—C13	1.377 (4)
N3—C12	1.474 (4)	C13—C14	1.364 (4)
N4—C14	1.478 (4)	C13—H13	0.9400
			
C1—O1—H1	108 (4)	C4—C5—C6	116.9 (3)
C5—O3—H3	107 (4)	C7—C6—C5	122.7 (3)
O4—N1—O5	125.1 (3)	C7—C6—N1	117.5 (2)
O4—N1—C6	118.3 (2)	C5—C6—N1	119.8 (3)
O5—N1—C6	116.6 (3)	C6—C7—C8	118.6 (3)
O7—N2—O6	125.3 (3)	C6—C7—H7	120.7
O7—N2—C8	117.8 (3)	C8—C7—H7	120.7
O6—N2—C8	116.8 (3)	C9—C8—C7	121.8 (3)
O9—N3—O10	125.2 (3)	C9—C8—N2	121.9 (2)
O9—N3—C12	117.4 (3)	C7—C8—N2	116.4 (2)
O10—N3—C12	117.4 (3)	C8—C9—C4	119.3 (3)
O11—N4—O12	125.1 (3)	C8—C9—C10	120.2 (3)
O11—N4—C14	116.7 (3)	C4—C9—C10	120.4 (2)
O12—N4—C14	118.1 (3)	O8—C10—C11	121.1 (3)
O1—C1—C14	119.3 (3)	O8—C10—C9	121.0 (3)
O1—C1—C2	123.4 (3)	C11—C10—C9	117.7 (3)
C14—C1—C2	117.2 (3)	C12—C11—C2	119.3 (3)
C11—C2—C1	120.1 (3)	C12—C11—C10	120.3 (3)
C11—C2—C3	120.3 (2)	C2—C11—C10	120.0 (2)
C1—C2—C3	119.6 (3)	C13—C12—C11	121.7 (3)
O2—C3—C4	120.5 (3)	C13—C12—N3	116.8 (3)
O2—C3—C2	120.2 (3)	C11—C12—N3	121.6 (3)
C4—C3—C2	119.3 (3)	C14—C13—C12	118.6 (3)
C9—C4—C5	120.6 (3)	C14—C13—H13	120.7
C9—C4—C3	120.5 (3)	C12—C13—H13	120.7
C5—C4—C3	118.9 (3)	C13—C14—C1	123.0 (3)
O3—C5—C4	124.4 (3)	C13—C14—N4	117.3 (3)
O3—C5—C6	118.6 (3)	C1—C14—N4	119.7 (3)
			
O1—C1—C2—C11	177.7 (3)	C5—C4—C9—C8	2.9 (4)
C14—C1—C2—C11	−2.4 (4)	C3—C4—C9—C8	−176.4 (3)
O1—C1—C2—C3	−3.7 (5)	C5—C4—C9—C10	−179.1 (3)
C14—C1—C2—C3	176.3 (3)	C3—C4—C9—C10	1.6 (4)
C11—C2—C3—O2	176.3 (3)	C8—C9—C10—O8	11.7 (5)
C1—C2—C3—O2	−2.3 (4)	C4—C9—C10—O8	−166.3 (3)
C11—C2—C3—C4	−2.8 (4)	C8—C9—C10—C11	−173.9 (3)
C1—C2—C3—C4	178.6 (3)	C4—C9—C10—C11	8.1 (4)
O2—C3—C4—C9	176.3 (3)	C1—C2—C11—C12	4.3 (4)
C2—C3—C4—C9	−4.5 (4)	C3—C2—C11—C12	−174.3 (3)
O2—C3—C4—C5	−3.0 (4)	C1—C2—C11—C10	−168.6 (3)
C2—C3—C4—C5	176.1 (3)	C3—C2—C11—C10	12.8 (4)
C9—C4—C5—O3	178.9 (3)	O8—C10—C11—C12	−13.8 (5)
C3—C4—C5—O3	−1.8 (4)	C9—C10—C11—C12	171.8 (3)
C9—C4—C5—C6	−2.6 (4)	O8—C10—C11—C2	159.0 (3)
C3—C4—C5—C6	176.7 (3)	C9—C10—C11—C2	−15.4 (4)
O3—C5—C6—C7	178.2 (3)	C2—C11—C12—C13	−3.0 (5)
C4—C5—C6—C7	−0.5 (4)	C10—C11—C12—C13	169.9 (3)
O3—C5—C6—N1	0.2 (4)	C2—C11—C12—N3	176.3 (3)
C4—C5—C6—N1	−178.5 (2)	C10—C11—C12—N3	−10.8 (4)
O4—N1—C6—C7	142.1 (3)	O9—N3—C12—C13	−67.5 (4)
O5—N1—C6—C7	−37.2 (4)	O10—N3—C12—C13	111.6 (4)
O4—N1—C6—C5	−39.8 (4)	O9—N3—C12—C11	113.1 (3)
O5—N1—C6—C5	140.9 (3)	O10—N3—C12—C11	−67.7 (4)
C5—C6—C7—C8	3.2 (4)	C11—C12—C13—C14	−0.2 (5)
N1—C6—C7—C8	−178.8 (3)	N3—C12—C13—C14	−179.6 (3)
C6—C7—C8—C9	−2.9 (4)	C12—C13—C14—C1	2.2 (5)
C6—C7—C8—N2	176.9 (3)	C12—C13—C14—N4	−179.7 (3)
O7—N2—C8—C9	76.1 (4)	O1—C1—C14—C13	179.0 (3)
O6—N2—C8—C9	−106.8 (3)	C2—C1—C14—C13	−1.0 (5)
O7—N2—C8—C7	−103.7 (3)	O1—C1—C14—N4	1.0 (5)
O6—N2—C8—C7	73.4 (3)	C2—C1—C14—N4	−179.0 (3)
C7—C8—C9—C4	−0.1 (4)	O11—N4—C14—C13	−38.1 (5)
N2—C8—C9—C4	−179.9 (3)	O12—N4—C14—C13	139.6 (4)
C7—C8—C9—C10	−178.1 (3)	O11—N4—C14—C1	140.0 (4)
N2—C8—C9—C10	2.1 (4)	O12—N4—C14—C1	−42.3 (5)

### Hydrogen-bond geometry (Å, °)

**Table:**

*D*—H···*A*	*D*—H	H···*A*	*D*···*A*	*D*—H···*A*
O1—H1···O2	0.84 (3)	1.84 (3)	2.579 (3)	146 (5)
O3—H3···O2	0.84 (4)	1.82 (3)	2.576 (3)	148 (5)
